# Clinical Experience with Off-Label Intrathecal Administration of Selected Antibiotics in Adults: An Overview with Pharmacometric Considerations

**DOI:** 10.3390/antibiotics12081291

**Published:** 2023-08-05

**Authors:** Anouk E. Muller, Peter van Vliet, Birgit C. P. Koch

**Affiliations:** 1 Department of Medical Microbiology, Haaglanden Medisch Centrum, 2512 VA The Hague, The Netherlands; 2 Department of Medical Microbiology and Infectious Diseases, Erasmus University Medical Center, 3015 GD Rotterdam, The Netherlands; 3 Center for Antimicrobial Treatment Optimization Rotterdam (CATOR), 3015 GD Rotterdam, The Netherlands; b.koch@erasmusmc.nl; 4 Department of Intensive Care Medicine, Haaglanden Medisch Centrum, 2512 VA The Hague, The Netherlands; p.van.vliet@haaglandenmc.nl; 5 Department of Hospital Pharmacy, Erasmus University Medical Center, 3015 GD Rotterdam, The Netherlands

**Keywords:** intrathecal administration, antimicrobial, intracerebral infection

## Abstract

Drain-associated intracerebral infections are life-threatening emergencies. Their treatment is challenging due to the limited penetration of antibiotics to the site of infection, resulting in potentially inadequate exposure. The emergence of multidrug-resistant pathogens might force the use of off-label intrathecal (IT) doses of antibiotics. We reviewed the literature on general aspects determining intrathecal dosing regimen, using pharmacometric knowledge. We summarised clinical experience with IT doses of antibiotics that are usually not used intrathecally, as well as the outcome of the cases and concentrations reached in the cerebrospinal fluid (CSF). Factors determining the IT regimen are the size of the ventricle system and the CSF drainage volume. With regard to pharmacometrics, pharmacokinetic/pharmacodynamic indices are likely similar to those in non-cerebral infections. The following number (N) of cases were described: benzylpenicillin (>50), ampicillin (1), ceftazidime (2), cephaloridine (56), ceftriaxone (1), cefotiam (1), meropenem (57), linezolid (1), tigecycline (15), rifampicin (3), levofloxacin (2), chloramphenicol (3) and daptomycin (8). Many side effects were reported for benzylpenicillin in the 1940–50s, but for the other antibiotics, when administered correctly, all side effects were minor and reversible. These data might help when choosing an IT dosing regimen in case there is no alternative option due to antimicrobial resistance.

## 1. Introduction

The incidence of community-acquired meningitis caused by multi-resistant micro-organisms is still low; however, these infections after neurosurgery might become more prevalent due to the overall rise in antibacterial resistance [1,2]. Infections after neurosurgery are also frequently complicated by the presence of intracerebral devices, such as external ventricular drains (EVD) and external lumbar drains (ELD), used in the treatment of elevated intracranial pressure, and may present a life-threatening emergency.

Both the blood–brain barrier and the blood–CSF barrier protect the central nervous system (CNS) from toxic compounds present in the systemic circulation. However, in the treatment of cerebral infections, both barriers complicate the achievement of adequate concentrations of antibiotics at the site of infection. Therefore, in some cases, extremely high intravenous doses have been administered, such as 24 g/24 h cefotaxime, 15 g/24 h meropenem or 8 g/24 h imipenem [3,4,5]. Another option to overcome this obstacle might be to bypass the blood–brain barrier by administering the antibiotics intrathecally. The term intrathecal (IT) is a general term including both intraventricular (IVT) and intralumbar (IL) injection in, respectively, the cerebral ventricles and the lumbar thecal sac.

Currently, the number of antibiotics that are frequently administered, intrathecally, is limited. Only polymyxin B is registered by the FDA, and the EMA recommends maximum IVT doses of colistin of 125,000 IU [6,7]. However, the increasing rates of antimicrobial resistance and the limited penetration of antibiotics through the blood–brain barrier (BBB), might force the use of IT administration of antibiotics that are usually not used intrathecally to achieve an effective concentration at the site of infection.

For several antibiotics, IT use has been reviewed [8,9,10]. The range of doses administered intrathecally is quite broad. For vancomycin, for example, IT doses reported in the literature range from 5 to 50 mg q24h [8]. This broad range indicates that potentially patient-related factors might be involved in determining the dose for an individual patient and that the dosing regimen might be optimised based on pharmacometric knowledge. This includes information on the pharmacokinetics at the site of infection and on the exposure–response relationship in the CNS. We therefore aimed to describe the factors involved in the designing of an IT dosing regimen in general and give an overview of available data on the IT use of antibiotics that are normally not administered via this route. Information on IT dosing of antibiotics more regularly used intrathecally can be found elsewhere [8,9,11,12,13,14]. 

## 2. Results

### 2.1. Physiological Factors Influencing Intrathecal Pharmacokinetics

**Figure 1:**
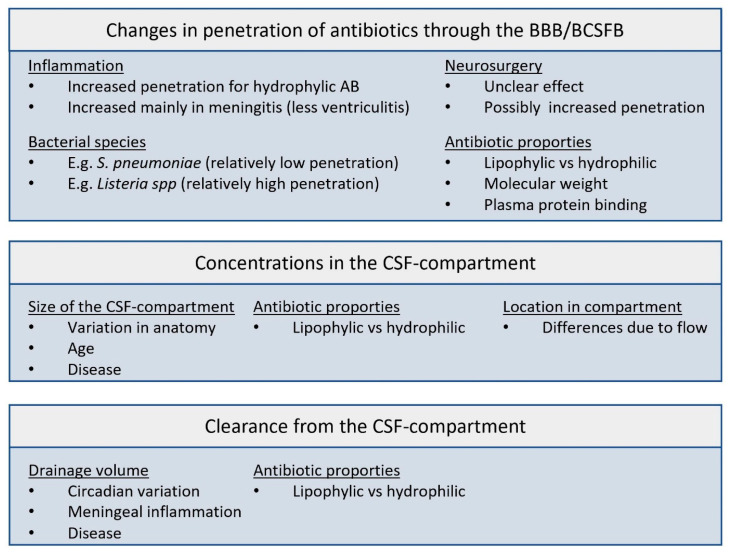
Factors involved in the pharmacokinetics of antibiotics in the CSF compartment. BBB: blood–brain barrier; BCSFB: blood–cerebrospinal fluid barrier; AB: antibiotic.

As antibacterial efficacy is dependent on a certain antibiotic exposure that needs to be reached at the site of infection, the amount of antibiotics reaching the CSF as well as the resulting concentration–time profiles in the CSF are of importance. Several factors might be taken into consideration in choosing a dosing regimen for IT administration. Figure 1 shows a summary of the factors involved.

#### 2.1.1. Blood–Brain Barrier and the Blood–CSF Barrier

Both the blood–brain barrier (BBB) and the blood–CSF barrier (BCSFB) are lipid layers surrounding the CNS aiming to protect the brain from toxic agents present in the systemic circulation. These barriers thereby limit the penetration of antibiotics to the site of infection when treating meningitis or ventriculitis. For example, the penetration of meropenem into the CSF in patients with (suspected) ventriculitis has a mean of only 9–18% [15,16], and a high interpatient variability has been reported. This high interpatient variability is in line with observations for vancomycin [17]. The low and unpredictable penetration into the CSF puts several patients at risk for insufficient exposure at the site of infection. 

Meningeal inflammation during infection leads to the opening of tight junctions in the BBB. The level of inflammation, which is more pronounced in meningitis as compared to that in ventriculitis might, for some antibiotics, lead to an increased penetration into the CSF [14,18]. This increased penetration mainly applies to hydrophylic antibiotics [19]. It has also been suggested that the BBB might be disrupted during neurosurgery, resulting in an increased penetration of systemically administered antibiotics. However, for vancomycin, it has been shown that the penetration in the CSF in patients after neurosurgery was similar to the penetration in patients with uninflamed meninges [20], making this statement questionable. Generally, though inflammation during infection and disruption of the BBB during surgery might both increase the potential penetration into the CSF, the true penetration into the CSF will still be highly variable and unpredictable.

From experimental animal models, it is also suggested that the infecting bacterial species might influence the permeability of the BBB. The presence of *Streptococcus pneumoniae* and *Escherichia coli* might result in a relatively low permeability, while the penetration into the CSF in infections caused by *Haemophilus* spp. and *Listeria monocytogenes* is higher [21,22]. For ceftriaxone, for example, the CSF/serum penetration was found to be 2.7% in case of a *S. pneumoniae* infection, while it was 9.0% in a *H. influenzae* infection in the same study [22]. Also, when the mezlocillin penetration into the CSF was compared between an infection with *E. coli* and *Listeria monocytogenes*, different values for the penetration were found of 6–11% and 16–20%, respectively [21].

#### 2.1.2. Size of the CSF System

The size of the CSF compartment is important to determine the volume of distribution for intrathecal dosing (V_CSF_). After penetrating the BBB, the size of the CSF compartment is one of the determinants for the concentrations reached locally. V_CSF_ consists of four ventricles, an aqueduct, basal cisterns and the subarachnoid space over the convexities and in the spinal cord. There is considerable interindividual variability in the size of the V_CSF_, depending on age, widths of the ventricles and cerebral subarachnoidal space, length and width of the spinal canal and underlying disease [8]. Several studies estimated the size of the CSF for healthy adults with mean values between 250 mL and 326 mL [23,24,25], patients with communicating hydrocephalus of mean 488 mL [25] and patients with non-communicating hydrocephalus of mean 593 mL [25]. The size of V_CSF_ might be reduced by the presence of blot clots in the ventricles of the basal cisterns.

Although the CSF compartment is convoluted, the distribution cannot be considered homogenous [8,26]. Overall, about two-thirds of the CSF is produced by the choroid plexus, and the remaining one-third originates from the extracellular space of the brain and spinal cord. The equilibration in the CSF is facilitated by the oscillations in the CSF based on the heartbeat and respiration. The net flow of the CSF is from the ventricles to the cisterna magna and from there to the cerebral convexities and into the spinal canal. As a result, antibiotic concentrations in CSF sampled by lumbar puncture might differ from those taken from an extraventricular drain.

Once the antibiotics reach the CSF, they can enter the extracellular fluid of the brain and spinal cord, since there is not a tight barrier between the CSF and extracellular fluid of nervous tissue. The diffusion from the CSF into the extracellular space of the brain and spinal cord occurs against the gradient of the CSF bulk flow (directed from nervous tissue into the CSF) [8].

#### 2.1.3. Location of Antibiotic Administration

As the CSF compartment is not homogenous and there is a net flow of CSF out of the ventricles, the location where the IT doses are administered makes a difference. Differences in concentrations can occur between the ventricular, cisternal and lumbar part of the CSF compartment [27]. As IT doses are most frequently prescribed to treat ventriculitis the antibiotic exposure needs to be optimal in the ventricles and the location of administration is therefore preferable intraventricular. For most drugs, achieved concentrations in the lumbar CSF are higher as compared to those in the ventricular CSF after IV administration [14]. Intraventricular dosing results in distribution throughout the CSF compartment, unless the system is blocked by, for example, bleeding. Due to the net flow of CSF out of the ventricles, antibiotic dosing intralumbar or intraspinal will result in lower concentrations in the ventricular CSF then after intraventricular dosing [28] and the concentrations will be unevenly distributed in the CSF space, more variable and might therefore be inadequate in the ventricles. Pharmacokinetic data therefore suggest that intraventricular dosing is preferred over intralumbar dosing, especially in the treatment of ventriculitis. However, clinically there is no evidence to support this [19]. 

#### 2.1.4. Drainage Volume

The drainage volume is the volume (in mL/day) that is eliminated from the CSF compartment per day. The drainage volume is therefore important for the clearance of antibiotics from the CSF. The external part of the drain is usually kept at a standard level, and the CSF fluid will be excreted when the intracranial pressure exceeds the pressure in the drain. Changing the level of the drain might therefore alter the drainage volume. For vancomycin as well as for meropenem, it has been shown that the drainage volume is a crucial factor for the determination of the IT dose [29,30,31]. 

The drainage volume also depends on the CSF production, which is not constant over time. Both in healthy volunteers and in patients with external ventriculostomies, the CSF production appeared to have a circadian variation with the minimum production of 12 ± 7 mL/h around 6 p.m. and the nightly peak around 2 a.m. of 42 ± 2 mL/h [32,33]. Meningeal inflammation might result in a reduced production of CSF, thereby limiting the drainage volume and the clearance of antibiotics from the CSF [14]. The timing of the IT dose might therefore also be of importance.

### 2.2. Other Factors Influencing the Intrathecal Pharmacokinetics

#### 2.2.1. Antibiotic Properties

The clearance of drugs from the CSF to the blood (CL_CSF to Blood_) depends on several processes, and these are of importance depending on the properties of the drugs. The processes important for the CL_CSF to Blood_ are as follows: 1. bulk flow (equals the CSF production rate); 2. retrograde diffusion across the blood–CSF barrier and the blood–brain barrier; and 3. active transport. Large hydrophylic molecules, such as aminoglycosides, are mainly cleared from the CSF by the CSF bulk flow. As a result, these drugs have a reduced clearance in hydrocephalic patients [8,34]. On the other hand, small moderately lipophilic molecules, such as quinolones, are mainly cleared through passive elimination via retrograde diffusion across both barriers [35]. 

Differences in the permeability of the BBB and the V_CSF_ are also partly explained by the difference in properties of hydrophylic or lipophylic antibiotics. The hydrophylic nature of antibiotics limits the penetration into the CSF. Their V_CSF_ equals the volume of V_CSF_ plus the fraction of the extracellular space of the brain that easily equilibrates with the CSF [36,37]. Since lipophilic drugs in the CSF equilibrate more easily with adjacent spaces and are able to bind to lipid membranes, the V_CSF_ of lipophilic drugs is usually larger as compared to hydrophylic drugs. Penetration through the BBB caused by inflammation during meningitis is increased for hydrophilic antibiotics, while for lipophilic antibiotics, this has a minimal effect [19]. 

Both molecular weight and protein binding also influence the penetration into the CSF. In the presence of an intact barrier, only the free fraction can penetrate into the CSF, since binding proteins pass the barriers only in a small degree [38]. For drugs with a low protein binding such as ceftazidime, the unbound fraction available for CNS penetration is therefore high. Protein binding in CSF is believed to be lower as compared to serum/plasma. For ceftriaxone, for example, serum/plasma protein binding is in the range of 80–95%, while in the CSF, it is found to be 18% ± 6% [39]. The molecular weight also influences the potential to penetrate the BBB, and drugs with large molecular weight, such as vancomycin and daptomycin, have a poor penetration.

#### 2.2.2. Simultaneous Systemic Dosing

**Figure 2:**
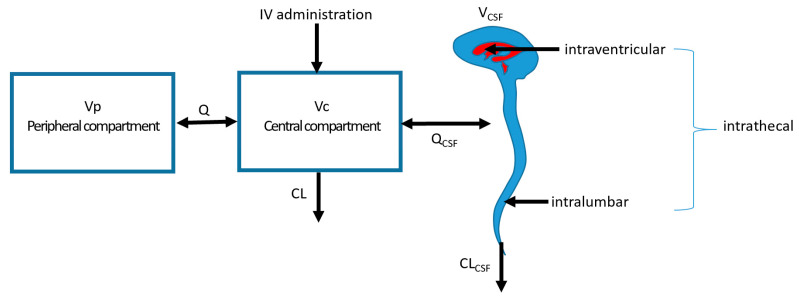
Multicompartment system with different locations of administration. Vp: peripheral volume of distribution; Vc: central volume of distribution; V_CSF_: volume of distribution of the CSF compartment; Q: intercompartmental clearance; CL: clearance; IV: intravenous; Q_CSF_: intercompartmental clearance between Vc and V_CSF_; CL_CSF_: clearance from the CSF compartment.

As the central volume of distribution is connected with the volume of distribution of the CSF concentrations in both compartments, they might influence each other (Figure 2). It is therefore important to consider the effect on the CSF concentrations after multimodal treatment using both IV and IT administration of antibiotics. Combined dosing of IV with IT administration is likely to result in slightly higher concentrations in the CSF. In a population PK model with meropenem in both serum and CSF, it has been shown that in order to achieve adequate CSF concentrations, relatively low IV doses of 500 mg q12h are needed for pathogens with MICs up to 4 mg/L and a drainage volume up to 200 mL/day [30]. This illustrates that regular IV dosing regimen are not always required simultaneously with the IT doses. However, it is questionable whether simultaneous IV dosing has a relevant effect on the PK in the CSF for all antibiotics [8]. Since most patients are treated with the combination of IV plus IT doses, there is not enough evidence to reduce the regimens to IT only.

### 2.3. Pharmacodynamics of Antibiotics in the Cerebrospinal Fluid 

The penetration of antibiotics into the CSF should be considered in the context that the principal determinant of the antibacterial efficacy is the antibiotic exposure related to the minimal inhibitory concentration (MIC) of the pathogen. Conclusions based on a single concentration in CSF after an IV dose can be incorrect, since the peak of the concentration–time curve in CSF is often delayed compared to the peak in serum. Ratios between CSF and plasma concentrations, frequently reported in the literature, can therefore be misleading. To best describe the penetration into the CSF the ratio between the area under the concentration–time curve (AUC)_CSF_ and AUC_serum_ or the %fT > MIC should be determined. After an intraventricular dose, the peak in the ventricular CSF will be reached immediately, but to describe distribution and clearance, multiple CSF concentrations are needed. As IT dosing is usually accompanied by IV dosing, the timing of the IV dose might also be of influence on the concentration–time profile in the CSF. In patients with an external ventriculostomy, CSF concentrations can be determined repeatedly, but sampling is limited by the fact that every manipulation of the ventriculostomy is an infectious risk.

Despite the fact that penetration into the CSF is best described by the AUC_CSF_/AUC_serum_ ratio, the interpretation of these penetration ratio values is not straightforward. For the selected antibiotics for which data on IT dosing are available, the values after IV administration are presented in Table 1. A low penetration ratio indicates that the amount of antibiotic entering the CSF is relatively limited. However, a low penetration ratio after IV dosing does not automatically mean that the antibiotic cannot be used in the treatment of meningitis. This is illustrated by the very low penetration ratio of ceftriaxone of 0.007 [40]. Ceftriaxone is frequently used to treat meningitis with IV dosing. This low penetration ratio is likely caused by the high serum protein binding of ceftriaxone [41], and also, the relatively low MIC values in the wild-type distribution of the pathogens might contribute to its effectivity in the treatment of meningitis. The penetration ratio by itself therefore cannot be used to decide whether an antibiotic is suitable in the treatment of meningitis after IV dosing or that additional IT doses are required. Other factors, such as the serum protein binding, the range in MIC values in the wild-type distribution of the pathogen and the pharmacodynamics, should also be taken into account [42].

To predict the antibacterial efficacy in the CSF, knowledge on the pharmacodynamics (PD) in the CSF is needed. For most antibiotics, the PK/PD index in serum is known; however, limited data are available on the PD in the CSF. Due to the restricted nutritional supply and acidic pH of the CSF, bacteria therein multiply less rapidly as compared to those in blood [8]. Also, because of the absence of complement and antibodies, the PD in CSF may differ from those in other body sites [19], but there is no indication that the concepts of time-dependent versus concentration-dependent efficacy are not applicable [19]. The PK/PD indices that are best correlated with efficacy should also be taken into account in the design of the dosing regimen.

The magnitude of the PK/PD indices correlated with antibacterial efficacy in the CSF is largely unknown, and the concentration–time profile of most antibiotics in the CSF is also unknown. To link concentrations to efficacy, a practical approach is used, correlating a single antibiotic concentration to the MIC or minimal bactericidal concentration (MBC) of the pathogen. A CSF concentration of at least 10 times that of the MIC or MBC has been correlated to the efficacy of beta-lactams and quinolones [54,55,56] and a pneumococcal meningitis model suggested a vancomycin-CSF-peak-to-MBC ratio >4 to be adequate [57]. The IDSA guideline recommend concentrations of 10–20× MIC [58].

Although little is known on the magnitude of the PK/PD indices needed for an antibacterial effect in the CSF, clinical data suggest an effect after IT dosing. In a study on the IT dosing of post-neurosurgical patients with meningitis (N = 30) or ventriculitis (N = 4) with persistent positive CSF culture despite IV treatment, the results showed an overall mean time to sterilise the CSF after IT administration of 2.9 ± 2.7 days (range of 1–12 days) [59]. In 50% of patients, the CSF cultures were negative <24 h, and in an additional 18%, they were negative within 48 h after the IT dose. The average time to sterilisation in the ventriculitis patients was 6.5 days.

### 2.4. General Aspects of IT Treatment

#### 2.4.1. Micro-Organisms

All micro-organisms have the potential to cause drain-associated infections. But, the most frequently reported micro-organisms are *coagulase-negative staphylococci* (especially *Staphylococcus epidermidis*), *S. aureus*, *P. acnes* and Gram-negative bacilli (including *Escherichia coli*, *Klebsiella species*, *Enterobacter species*, *Citrobacter species*, *Serratia species*, *Acinetobacter* spp. and *Pseudomonas aeruginosa*) [58,60].

*A. baumannii* is an important pathogen in this patient category. Of all healthcare-associated meningitis, 3.6–11.2% of cases are caused by *A. baumannii* [13]. It is a difficult-to-treat pathogen, for which the IDSA guidelines even recommend treating meningitis or ventriculitis with a combination of IV and IVT treatment with polymyxins [58]. 

The distribution in the MICs between various species for a specific antibiotic can be quite different. Even for a single antibiotic–pathogen combination, there is a considerable amount of variation that needs to be taken into account when determining the MIC [61]. Therefore, the epidemiological cut-off value (ECOFF) is usually taken into account when designing a dosing regimen for susceptible pathogens or when performing therapeutic drug monitoring. This ECOFF value is the highest value of the wildtype distribution and can be found on the EUCAST website [62].

#### 2.4.2. Therapeutic Drug Monitoring

The choice whether to perform therapeutic drug monitoring (TDM) or not depends on several factors. There needs to be information on how to interpret the concentration. Interpretation of the concentration in the CSF is complex, since very little is known regarding the PD and the target concentrations. In a retrospective cohort study of 105 patients with vancomycin or an aminoglycoside administered via IVT, there is a higher proportion of the survivors that had TDM on CSF concentrations as compared to the non-survivors [11], suggesting a potential benefit of TDM.

On the other hand, it could be used to avoid toxicity by detecting extremely high concentrations, although CSF concentrations associated with toxicity are also not known. For vancomycin, for example, serious toxicity and adverse events do not appear to be correlated with the CSF concentration [63]. Systematic monitoring of CSF concentrations and correlating them with neurotoxicity could increase our knowledge and predict toxicity in the future. In general, it could be useful to detect IT doses that are administered as bolus or as short infusions and to determine the concentration–time profile in individual patients when multiple samples are needed.

#### 2.4.3. Clinical Outcome

To study the clinical outcome after IT administration compared to IV administration of antibiotics, comparative studies are needed. Obviously, for antibiotics that are not frequently used with IT administration, those studies are not available. Only for meropenem is there one retrospective study in combination with vancomycin [64], showing the beneficial effect of IL administration as compared to IV only. A review on cephalosporin cephaloridine concluded that IT dosing had a positive effect on the outcome in the treatment of bacterial meningitis [65]. 

Only for colistin 2 has a meta-analysis been performed. In 2019, Hu et al. published a review with a meta-analysis to compare IT/IVT administration with IV administration in patients with post-neurosurgical intracranial infection due to multidrug-resistant Gram-negative bacteria [9]. The vast majority of the studies included in the meta-analysis for the outcome ‘mortality’ included the use of colistin. The use of IT/IVT antibiotics was associated with a lower risk on mortality (nine studies were included; pooled OR 0.15; CI 0.08–0.28; *p* < 0.001; pooled number of deaths: in the IVT group, 22 of 127 cases, and in the IV group, 95 of 152 cases) and with a high microbiological clearance rate (two studies were included; pooled OR 0.02; CI 0.01–0.1; *p* < 0.001; pooled number of bacterial clearance: in the IVT group, 30 of 32 cases, and in the IV group, 10 of 47 cases.) [9]. Another meta-analysis was performed on IT plus IV colistin versus IV colistin only for *Acinetobacter baumannii* infections in post-neurosurgical patients [66]. They included five studies, and four of those were also included in the meta-analysis of Hu et al. The overall conclusion was that patients treated with a combination of IT and IV colistin had an 84% lower risk of dying due to an *A. baumannii* infection [66]. 

Gower et al. published a case series of 39 adults with a Gram-negative bacillary meningitis [67]. The study included various micro-organisms, but also many different antibiotic regimen. IT gentamicin, tobramycin, amikacin, polymyxin B and cefamandole were used. The overall mortality was 36%, and when the groups were divided into subgroups based on IT therapy, the mortality rates were as follows: 36.4% in patients without IT therapy (N = 22), 37.5% in patients with a short course of IT therapy (N = 8) and 26.6% in patients with a full course of IT therapy. Although this is in line with the results of Hu et al., the results must be interpreted with caution since there is no detailed information on the antibiotic combinations nor on the reasons behind the choice of a specific regimen [67]. 

Although several studies reported a beneficial effect of IT administration of antibiotics on the outcome as compared to conventional dosing, there is also a study on IT gentamicin in 52 children, reporting an increased mortality rate in the IT group [9,63,67,68]. Another study found that for carbapenem-resistant isolates the outcome was better in the patient group treated with IT dosing, but this beneficial effect was not found for other micro-organisms [69]. This is in line with the studies on the polymyxins, which are usually prescribed in the treatment of carbapenem-resistant Gram-negatives. This underlines the previous conclusion that IT dosing should only be used in patients when there is no reliable alternative.

In general, studies on clinical outcome compare standard treatment to IT dosing. Since the group of patients that is treated for meningitis or ventriculitis is severely ill, this might result in larger variability in PK than usual [70]. For the standard IV dosing regimens used, there might therefore be a risk of underdosing. When patients with the standard IV regimens are indeed underdosed, this might increase the difference in outcome with IT dosing, while part of the difference might be explained by underdosing of the IV group. The actual benefit of IT dosing could therefore be smaller than that reported in studies. 

#### 2.4.4. Adverse Events and Risks of IT Administration

Most reported side effects of IT administration are chemical ventriculitis or meningitis, seizures or local adverse events or infection [60,71]. A meta-analysis including 23 studies (229 patients) reported that the overall complication rate was 13%; chemical meningitis and seizures represented the majority of the complications, with an occurrence rate of 11% and 7%, respectively [71]. However, since patients in the need for IT treatment are usually very sick, there might be underreporting of side effects. On the other hand, side effects as described for IT dosing have also been described after IV administration only. For colistin, the prevalence of side effects after IT was similar to those after IV dosing [72].

Neurotoxic side effects of beta-lactam antibiotics are known to occur when there is high exposure what can be caused by very high doses or in the presence of renal failure. They can induce confusion, encephalopathy, myoclonus and epileptic seizures particularly in patients with underlying neurological disorders [73,74]. The potency to induce seizures is relatively high for cefazolin, cefepime, benzylpenicillin and imipenem, whereas this is much lower for ampicillin, ceftazidime and meropenem [74]. 

An important side effect of IT dosing is chemical ventriculitis or meningitis, usually mild and reversible. Clinically, it resembles bacterial meningitis, presenting with fever, altered mental state, elevation of white blood cell count in the CSF and low glucose concentrations [13]. It is difficult to distinguish a chemical reaction on an antibiotic from the reappearance of signs of meningitis, which can be caused by a relapse of the existing infection or an infection with a new pathogen due to the multiple manipulations [13].

The IT administration of antibiotics often involves a drain system. Every manipulation of the system increases the risk on drain/device infection. Though the antibiotics might be indicated for a specific pathogen, there is a risk of introducing another pathogen through manipulation of the system, such as irrigation [75]. Therefore, preparation of the antibiotics administered intrathecally must be performed under sterile circumstances, and precautions must be taken to avoid causing an infection.

### 2.5. Clinical Experience with IT Administration of Selected Antibiotics

The literature search revealed for most antibiotics only case reports. Only for meropenem 2 population pharmacokinetic are models available. The data found will be described below. For fosfomycin, ciprofloxacin, moxifloxacin, piperacillin, cefotaxime, imipenem, ertapenem, metronidazole, co-trimoxazole, tetracycline and doxycycline, no data were found. Data for the cases of several antibiotics are shown in Table 2.

#### 2.5.1. Penicillins

In the 1940s and 1950s, the use of IT penicillin was quite common. In 1941, it was concluded that IT administration of penicillin was safe, as after the administration of penicillin in the cisterna magna of rabbits there were no histological disturbances [86]. The usual dose for adults was 6 mg (10,000 units) [87], and it was used for pneumococcal meningitis, which used to be a fulminating and rapidly fatal disease in those days [88]. Many case reports have been published describing the serious side effects of IT administration of penicillin, such as generalised flaccid paralysis, flaccid paraplegia or death [89,90,91,92]. In several cases, doses administered intrathecally were higher than the usual dose of 10,000 unit, as described by Wood [87]; the described effects are very serious, but it was also mentioned that the pneumococcal meningitis itself might in part contribute to the side effects. Sweet et al. described 16 patients with pneumococcal meningitis, 9 of whom died, and of the other 7 patients, the side effects of IT penicillin were reported in 4 patients [91]. Due to the side effects and the optimized IV dosing regimen, IT administration of penicillin is no longer used.

One case on the use of IT ampicillin [84] is described in the literature of a patient with *E. coli* meningitis. He started with a regimen of 20 mg q12h IT ampicillin with 500 mg q6h oral doses. But, due to microbiological failure the IT doses were increased to 40 mg q12h. The patient was cured and experienced no side effects.

#### 2.5.2. Cephalosporins

Several cephalosporins, such as ceftriaxone and ceftazidime are commonly used in the treatment of bacterial meningitis and are usually not administered intrathecally. There are some data on the IT administration of cephaloridine, a first-generation cephalosporin that is no longer available for therapeutic use possibly due to renal toxicity. Furthermore, two cases on the use of ceftazidime have been described, as well as two cases in which the cephalosporin was dosed extremely high after an error in the preparation of the solution or the administration [65,85,93].

In 1975, a review was published on the use of cephaloridine [94]. They found that patients receiving concomitant IT and IV cephaloridine (N = 56) responded significantly better (*p* < 0.005) in the treatment of bacterial meningitis, as compared to those treated only with IV cephaloridine (N = 16). The IT doses administered varied from 5 to 100 mg/day. 

Two cases have been described who were treated with intraventricular ceftazidime in doses of 10–20 mg twice per week. One patient with *P. aeruginosa* meningitis, who was treated as an out-patient for nearly two years, died after an attempted withdrawal of the intraventricular treatment [85]. The other patient was cured from a ventriculitis with an unknown pathogen after 16 IT doses of ceftazidime. Both patients did not have irreversible side effects [85]. 

Furthermore, there are two case reports on accidental high doses via IT administration of cephalosporins. In the first case, 800 mg ceftriaxone was administered intrathecally as a consequence of a dilution error (instead of the intended 8 mg) [93]. A solution of 50 mg ceftriaxone/mL was injected in a 74-year-old patient treated for pneumococcal meningitis and pansinusitis. After the injection, the patient experienced severe burning pain in the lumbosacral region radiating into both extremities. Since the pain did not respond to analgesics, 240 mL of CSF was exchanged. At 1 h after the injection, the CSF concentration was 4387 mg/L, and after the exchange of 240 mL CSF, the concentration decreased to 3384 mg/L. There were no permanent side effects. The second case was in a 66-year-old patient accidentally receiving 1.5 g cefotiam via an intrathecal port system put in place for pain management [65]. After the injection, the patient experienced muscular cramps, abdominal pain, massive general myoclonic jerks, massive pain and dyspnoea. Approximately 20 h after the injection, the CSF concentration was 198.8 mg/L. On day 5, the CSF concentration was 10.1 mg/L. The patient was intubated and dialysed, but afterwards, he returned to his previous neurological state, with no signs of permanent damage due to the cefotiam.

#### 2.5.3. Carbapenems

Meropenem is frequently used to treat meningitis with a regimen of 2 g q8h. However, this IV regimen might not be sufficient for infections caused by difficult-to-treat micro-organisms. Therefore, the dosing regimen is sometimes increased up to even 15 g meropenem per day [4]. The need for a higher dosing regimen of meropenem in the treatment of Pseudomonas aeruginosa cerebral infections was also suggested by Konig et al. [95], who based their recommendations on a PK/PD model and a target in the CSF of 100% *f*T > 2× ECOFF of *Pseudomonas aeruginosa*. Instead of very high IV doses, IT administration might be an option. For meropenem, three studies are available. One is a retrospective study comparing the clinical outcome and the occurrence of side effects of vancomycin and meropenem [64]. Two studies determined concentrations in CSF as well as in plasma and analysed the data using population pharmacokinetic modelling [30,31]. In the retrospective study, 86 patients were included with an infection after cranial trauma surgery [64]. Two groups were compared. In both groups CSF was released by lumbar cistern drainage, and the control group received vancomycin and meropenem IV, whereas the experimental group was treated with vancomycin and meropenem intrathecally (20 mg q12h) via the drain. The recovery rate in the experimental group was 95% versus 72% in the control group (*p* < 0.05). Also the cure time was lower in the experimental group (11 ± 5 days and 23 ± 9 days for the experimental and control group, respectively; *p* < 0.001). Side effects occurred less in the experimental group (*p* < 0.05). While these dosing regimens are frequently used in clinical practice, vancomycin is usually guided based on TDM. This study did not mention the use of TDM. Since this patient group of the critically ill is known to have a large variability in the PK, the possibility of underdosing should be taken into account in the interpretation of the data. No data were found on imipenem and ertapenem. 

The two other studies measured concentrations in plasma as well as in the CSF and analysed the data with a population pharmacokinetic analysis to be able to describe the time course over time, find important covariates and use the population model to predict the most optimal dosing regimen [30,31]. In both studies, a group of patients (9 patients with an aneurysm and 15 patients after neurosurgery) meropenem was dosed at 1990 mg IV q12h and 10 mg IT q12h, and after the intrathecal dose, the drain was clamped for 15 min. Meropenem was administered via IL injection in the first study and via IVT injection in the second study. An important factor that determines the exposure to meropenem in the CSF is the drainage volume per day. Based on simulations with the final model of the first study, the IV doses needed to be increased in patients with a drainage volume of >250 mL/day [31] for regimens designed from MIC values of 4 mg/L and 8 mg/L. In the second study, an increase in the IV as well as in the IVT dose was recommended for patients with a drainage volume of 200–300 mL/day and an MIC of 4, 8 or 16 mg/L and a second increase in both dosages in patients with a drainage volume of 300–400 mL/day [30]. Dosing regimens suggested are based on a target value of 100% fT > MIC. The suggested regimens are quite complex and based on mathematical modelling. Overall, MIC values of micro-organisms that are reported susceptible to meropenem using EUCAST breakpoints [96] will include MIC values up to 4 mg/L. Assuming, based on available data, that IT doses of 10 mg meropenem are well tolerated, and based on the pharmacokinetic models, an overall dosing regimen of 2 g IV and 10 mg IT q12h might be an option. In case the aim is to treat micro-organisms with MIC values higher than the wild-type distribution, especially in patients with a drainage volume > 200 mL/day, higher doses might be needed. Given the elimination rate of meropenem, an IT dosing regimen of q24h is likely not feasible. In both studies, a high variability between concentrations in the CSF was found. 

For meropenem, it is also important to mention the study of Hosmann et al. [97]. This study highlighted the importance of measuring concentrations at the site of infection. They measured meropenem concentrations after IV dosing in plasma, CSF as well as in the cerebral tissue via microdialysis. Concentrations in cerebral microdialysate were more than three times higher than those in CSF, showing that measuring CSF concentrations would highly underestimate brain tissue concentrations. Since the magnitude of the PK/PD indices needed for bacterial efficacy might also differ between an infection in the CSF and in brain tissue, it is not possible to interpret these values. The study clearly shows that designing optimal dosing regimen to treat intracerebral infection is very complex.

#### 2.5.4. Linezolid

Little is known on the use of linezolid for the treatment of intracerebral infections. It has a bioavailability of 100%, and penetration in the central nervous system is variable, ranging from 28 to 70% [98]. Since this is relatively high [99], linezolid is normally not administered intrathecally.

Only one case report on IT administration has been described in the literature, and concentrations in the CSF were not determined [80]. The IT doses of 10 mg were administered once daily in a concentration of 2 mg/mL for 15 days in total. No side effects were reported.

#### 2.5.5. Tigecycline

Tigecycline is currently approved for three indications: complicated skin and skin structure infections, complicated intra-abdominal infections and community-acquired bacterial pneumonia. Within the label, there is a box warning that there was an unexplained increased all-cause mortality in patients treated with tigecycline as compared to the comparators in a meta-analysis of clinical trials. It should therefore be reserved for use in situations when alternative treatments are not suitable [100].

Due to the poor BBB permeability of tigecycline, it is not recommended in the treatment of intracerebral infections with IV administrations. Tigecycline CSF concentrations after the usual 100 mg IV dose per day are only 0.035–0.048 mg/L, and increasing the daily dose to 200 mg is not well tolerated [101,102]. Several case reports (N = 14) have been described in the literature of IVT tigecycline in the treatment of infections caused by *A. baumannii*, *K. pneumoniae* and *K. oxytoca* (Table 3) [103,104,105,106,107,108,109,110,111,112,113,114,115]. The dosing regimen using IVT ranged from 1 mg q12h to 10 mg q12h. About half of the regimen used an IVT tigecycline dosing of q24h. In only two patients were concentrations determined in the CSF [103,112]. In the first patient, several concentrations were determined after an IVT dose of 1 mg and after a 2 mg IVT dose. The AUC_0–12h_ was calculated to be 230 h·mg/L and 1132 h·mg/L after the 1 mg and 2 mg IVT dose, respectively. Based on the reported concentrations in another patient after a 5 mg IVT dose, the AUC_0–12h_ can be estimated to be 887–1166 h·mg/L [112]. The magnitude of the PK/PD index correlated with bacteriostasis in a neutropenic thigh infection mouse model for E. coli and K. pneumoniae is a *f*AUC/MIC of median 5–6 h·mg/L [116]. As the AUCs in the CSF reached in these two patient are much higher than the PK/PD target value, high IVT doses might not be necessary. However, in both patients, the tigecycline was cleared from the CSF quite rapidly, resulting in undetectable or very low concentrations 12 h after the dose. It seems therefore to indicate the administration of IVT doses at q12h. Some patients failed on the initial regimen and were cured after the IVT dose was increased: in one patient, the dose was increased from 3 mg q24h to 4 mg q12h [104]; in the second patient, from 2 mg q24h to 2 mg q12h [105]; and from 2 mg q12h to 4 mg q12h in the third case [110].

In four cases, potential side effects were reported [105,110,111,113]. As tigecycline is used in these cases for the treatment of multidrug-resistant micro-organisms, it is often difficult to determine whether the observed side-effect is attributable to the tigecycline or to other co-administered drugs. In one case, a ventriculitis and a holocord myelitis were reported [105]. This patient also received colistin via IVT injection, and since this is a known side-effect of IVT colistin, this could well be caused by the colistin [13,117]. This is supported by the fact that in this patient, the IVT administration of colistin and tigecycline were stopped, and because the meningitis reoccurred, IVT tigecycline was restarted after one day, without further problems. One patient had a myoclonic seizure 8 h after the first IVT tigecycline dose [110]. This was treated, and due to a lack of alternative antibiotic options, the IVT tigecycline was continued while the patient used maintenance doses of phenytoin. 

Without further seizures, the patient completed the IVT tigecycline treatment of 14 days. For two other patients receiving IVT doses of 5 mg, the potential side effects were described: spinal arachnoiditis (after nine IVT doses); and reduced liver function after 7 days of IVT treatment [111,113].

#### 2.5.6. Rifampicin

Rifampicin is used as part of the combination regimen in the treatment of tuberculosis or as additive antibiotic to flucloxacillin or vancomycin to increase penetration in a biofilm. Two cases have been described in which rifampicin was switched to IT administration because of the failing efficacy of IV-administered combinations of antibiotics in the treatment of intracerebral tuberculosis infections (Table 2) [81,82]. The targeted concentration in the CSF was 15 mg/L [81]. In both cases, IT doses of 5 mg were used without side effects. In addition, Senbaga et al. described a case in which a 41-year-old patient accidentally received a dose of 600 mg rifampicin infusion intrathecally over 4 h [83]. The intention was to administer vancomycin IT and rifampicin IV, but the infusion systems were swapped. No adverse events occurred in this case. It is noteworthy to mention that intravenous preparations of rifampicin have trace doses of formaldehyde [83].

#### 2.5.7. Quinolones

Quinolones are known to have a potent seizure-inducing activity [118,119]. Overall, the incidence of central and peripheral nervous system reactions is estimated to be 0.9–2.1% [118]. A history of epilepsy, cerebral trauma and alcohol abuse are risk factors. Though severe reactions, such as hallucinations, depression and even convulsive seizures, are rare, intraventricular use of quinolones might not be a tempting option. But, since fluoroquinolones enter the CSF readily, IT use might not be necessary.

Two cases of intraventricular administration of levofloxacin have been described and summarised in Table 2 [78,79]. In both cases, patients were treated with an antibiotic regimen consisting of several drugs to treat meningitis due to multidrug-resistant *Mycobacterium tuberculosis*. Intraventricular doses of 1–2 mg per dose were administered for a prolonged duration without causing serious side effects. In one case, the levofloxacin dose was calculated assuming a CSF volume of 120 mL and a target CSF concentration of 8–10 mg/L [78]. During the first 1–2 months of treatment, there were minor adverse events ascribed to the levofloxacin: insomnia, myalgias and arthralgia. These later subsided [78]. Both patients were cured after months of treatment, but due to the regimens containing several antibiotics, it is unclear whether this is attributable to the intraventricular administration of levofloxacin.

#### 2.5.8. Chloramphenicol

Chloramphenicol is an antibiotic that is little used in industrialised countries, but it is included in the WHO list of essential medicines to be used for several indications, such as meningitis [120]. Three cases were reported in the literature on IT use of chloramphenicol [76,77]. After IT administration, chloramphenicol sodium succinate will be hydrolysed to the microbiologically active chloramphenicol in the ventricular fluid. There is a considerable difference in the doses used in the first two cases in 1951 (up to 750 µg) [76] and the more recent case in 2005 (25 mg/day) [77]. The accompanying systemic doses were also different (12 g oral vs. 3 g IV), and in the recent case, no concentrations in the CSF were measured. Furthermore, besides the two cases Anderson et al. described in detail (Table 2), they also mentioned that several patients were treated with 3 mg administered into the lumbar theca for at least one week [76]. But, no further details on accompanying systemic therapy or CSF concentrations were given on these patients. In the cases reported in 1951, chloramphenicol was administered in pure crystalline form as an IT solution at a concentration of 100 µg/mL [76]. In the recent case, the formulation used was not mentioned. Due to the differences and incomplete information, an overall conclusion with regard to the IT doses therefore cannot be drawn. 

In all three cases that were described in detail, no side effects were reported (Table 2). But, in the group of patients receiving 3 mg into the lumber theca, several side effects were noted: all patients reported depression with tearfulness, and patients with pre-existing cerebral tremor experienced marked accentuation of the tremor [76]. Both side effects disappeared with cessation of IT therapy.

#### 2.5.9. Daptomycin

Daptomycin is a cyclic lipopeptide that is used to treat infections due to Gram-positive micro-organisms. After systemic administration, daptomycin penetrates poorly into the CSF compartment, due to its low lipophilicity, significant protein binding and large molecular weight, thus resulting in insufficient concentrations to treat intracerebral infections [52,121]. In total, eight cases have been described in the literature with IT administration of daptomycin in the treatment of intracerebral infections due to Enterococci or Staphylococci (see Table 4) [122,123,124,125,126,127,128,129]. The IT doses administered ranged from 2.5 mg to 10 mg per dose, with the dosing frequency ranging from once per day to once per 72 h. The dose of 5 mg was used most frequently. For seven out of the eight cases, a positive outcome was reported. The patient in the eighth reported case relapsed and needed a second (extended) period of treatment before he was cured [125]. No serious side effects were reported in these eight cases.

## 3. Discussion, Overall Conclusions and Recommendations

Intrathecal administration of the antibiotics described in this review is off-label and is limited to those patients for whom clinicians run out of therapeutic options. When used without administration/preparation errors, and with the exception of benzylpenicillin, the reported side effects are generally mild and reversible. As side effects have been reported after both IV and IT administration, and due to the lack of clear correlation between CSF concentrations and toxicity, it could be questioned whether the administration of extremely high IV doses puts patients at lower risk of side effects compared to IT administration. It is possible that the CSF concentration that is aimed for causes a toxic effect regardless of the route of administration. 

With the current limited knowledge of PK/PD in the CSF, it is not possible to choose an evidence-based efficacious dosing regimen, and also, routine TDM is not recommended in the literature. Generally, for lipophilic drugs with a molecular weight > 1000 g/mol and hydrophylic drugs with a molecular weight > 400 g/mol, once-daily IT dosing is usually performed [8]. For antibiotics for which there is experience with multiple-dosing regimen, the highest regimen can be used in case of a high drainage volume, a pathogen with a relatively high ECOFF and/or a large distribution of the CSF. All available data are summarised in Table 5. Clamping of the drain is necessary to make sure that the administered antibiotic is distributed over the CSF space. The duration of clamping depends on the intracranial pressure and the tolerance of the patient.

## 4. Methods

A search was conducted in PubMed using the name of the individual antibiotic in combination with ‘intrathecal’ or ‘intraventricular’. References from the papers included were also checked to find missing references. The antibiotics selected were based on those mentioned in the review paper of Nau et al. [8] as antibiotics are usually not administered intrathecally. Included in the final search were the following: benzylpenicillin, ampicillin, piperacillin, cefuroxime, cefotaxime, ceftriaxone, ceftazidime, imipenem, meropenem, ertapenem, ciprofloxacin, levofloxacin, moxifloxacin, co-trimoxazole, linezolid, metronidazole, chloramphenicol, rifampicin, fosfomycin, doxycycline and tetracycline.

## Figures and Tables

**Table 1:** AUC_CSF_/AUC_serum_ ratios for the selected antibiotics.

Antibiotic	AUC_CSF_/AUC_serum_ Ratio in Uninflamed to Mildly Inflamed Meninges	AUC_CSF_/AUC_serum_ Ratio in Inflamed Meninges	References
Benzylpenicillin	-	-	-
Amoxicillin		0.058	[43]
Cephaloridine	-	-	-
Cefotiam	0	-	[44]
Ceftriaxone	0.007	-	[40]
Ceftazidime	0.054	-	[45]
Meropenem	0.47; 0.21; 0.25	0.39	[14,46]
Linezolid	0.53	-	[47]
Tigecycline	0.11	-	[48]
Rifampicin	0.22	-	[49]
Levofloxacin	0.71	-	[50]
Chloramphenicol	0.6–0.7	0.6–0.7	[14,51]
Daptomycin	-	0.008; 0.45	[52,53]

AUC: area under the curve; CSF: cerebrospinal fluid.

**Table 2:** Cases with IT doses of miscellaneous antibiotics.

Year (Ref.)	Antibiotic	Age/Sex	Diagnosis	Pathogen (ECOFF)	IT Dose	Number IT Doses	Accompanying IV Dose of Same AB	Concentration in CSF	Outcome (M/C)	Side Effects
1951 [76]	Chloramphenicol	44 y/M	Meningitis	*S. aureus* (16 mg/L)	Increasing from 100 to 750 µg q24h IT	19	2 g q6h oral	400 µg D: 10 mg/L; 500 µg D: 20 mg/L; 600 µg D: 30 mg/L and 750 µg: 40 mg/L	C	None
1951 [76]	Chloramphenicol	47 y/M	Meningitis	*S. aureus* (16 mg/L)	600–750 µg q24h IT	6	2 g q6h oral	40 mg/L	C	None
2005 [77]	Chloramphenicol	46 y/M	Meningitis	*E. faecium* (32 mg/L)	25 mg/day	35	3 g q24h IV	n.a.	C	None
2001 [78]	Levofloxacin	25 y/M	Meningitis	*M. tuberculosis* (n.a.)	1–1.5 mg q48h	90	500 mg IV	1 mg D: 0.38 mg/L (+2 h) and 0.57 mg/L (+6 h). 1.5 mg D: 1.64 mg/L (+6 h)	M, C	Insomnia, myalgia, arthralgia during first 1–2 months
2009 [79]	Levofloxacin	39 y/M	Meningitis	*M. tuberculosis* (n.a.)	1.5–2 mg First ~1.5 mo: 3×/wk ~4.5 mo 2×/wk ~3 mo: 1×/wk	~66	750 mg oral	1.5 mg D: 9.16/11.36 mg/L (+2 h); 2.06/ND (+6 h); 0.19/1.41 mg/L (+24 h). ND/ND (+48 h)	M, C	none
2016 [80]	Linezolid	31 y/F	Ventriculitis	*E. faecalis* (4 mg/L ^1^)	10 mg q24h	15	600 mg q12h oral	n.a.	M, C	none
1981 [81]	rifampicin	23 y/?	Meningitis	*M. tuberculosis* (n.a.)	5 mg q24h for 7 days; then 3 mg q48h	~34	n.a.	n.a.	Clinically improved	none
1992 [82]	Rifampicin	59 y/M	Meningo-encephalitis	*M. tuberculosis* (n.a.)	5 mg q24h	50	600 mg q24h IV	n.a.	C	none
2005 [83]	rifampicin	41 y/?	Postoperative infection after spinal decompression	n.a.	600 mg infused over 4 h IT (accidental)	1	no	n.a.	n.a.	none
1962 [84]	Ampicillin	17 y/M	Meningitis	*E. coli* (8 mg/L)	20 mg q12h 40 mg q12h	10 32	500 mg q6h oral	Ranging between 1.8 and 18.8 mg/L (8 of 12 measurements ≤8 mg/L)	M, C	none
1992 [85]	ceftazidime	70 y/M	Meningitis	*P. aeruginosa* (8 mg/L)	10 mg 2×/wk 15 mg 2×/wk	104 n.a.	no	n.a.	Patient died of infection 2 y after start of IT therapy	None for 10 mg doses Psychological changes for the 15 mg doses
1992 [85]	ceftazidime	26 y/M	Ventriculitis	unknown	20 mg 2×/wk 20 mg 1×/wk	8 8	no	n.a.	C	none

n.a.: not available; ND: not detectable; mo: month; D: dose; M: microbiological cure, C: clinical cure. IT: intrathecal; AB: antibiotic; CSF: cerebrospinal fluid; M: male; F: female. Doses were administered via IVT injection, unless stated otherwise. ^1^: no official ECOFF; values of 4 mg/L can be expected based on available distributions.

**Table 3:** Cases with IT tigecycline.

Year (Ref.)	Age/Sex	Diagnosis	Pathogen (ECOFF)	IT Dose	Number of IT Doses	Accompanying IV Dose of Same AB	Concentration in CSF	Outcome (M/C)	Side Effects
2016 [103]	67 y/M	Meningitis	*K. pneumoniae* (2 mg/L)	1 mg q12 or q48h; 2 mg dose (final one)	4 1	49 mg q12h; 48 mg q12h	1 mg D: AUC_0–12h_: 230 h·mg/L; 2 mg D: AUC_0–12h_ 1132 h·mg/L	n.a.	none
2017 [104]	50 y/M	Intracranial infection	*A. baumannii* (0.5 mg/L)	3 mg q24h (1 h closed system after administration); M failure dose increased to 4 mg q12g	6 days 3 mg q24h, 6 days high dose	50 mg q12h	n.a.	M: 3 days after start high dose. C	none
2017 [105]	22 y/M	Meningitis	*A. baumannii* (0.5 mg/L)	2 mg q24h (drain closed 2 h); 2 mg q12h; Relapse of meningitis: 4 mg/day restarted	45 days Relapse: 1 month	100 mg q12h	n.a.	M: after month of treatment of relapse. 12-month follow-up pt cured	Ventriculitis and holocord myelitis (also colistin IT)
2017 [115]	45 y/M	Meningitis	*A. baumannii* (0.5 mg/L)	10 mg q12h IL (drain closed 2 h)	12	no	n.a.	M, C	none
2018 [106]	70 y/F	Ventriculitis	*A. baumannii* (0.5 mg/L)	2 mg q12h	20	50 mg q12h	n.a.	M, C	none
2018 [107]	55 y/F	Ventriculitis + meningitis	*A. baumannii* (0.5 mg/L)	4 mg q24h (drain closed 4 h)	15	100 mg bid	n.a.	C	none
2018 [107]	50 y/M	Postoperative intracerebral infection	*A. baumannii* (0.5 mg/L)	4 mg q24h (drain closed 4 h)	15	no	n.a.	Discharged to rehab centre	None
2018 [107]	48 y/M	Ventriculitis + meningitis	*K. pneumoniae* (2 mg/L)	4 mg q24h (drain closed 4 h)	9	100 mg bid	n.a.	Discharged (ICU to ward)	none
2018 [108]	67 y/M	Ventriculitis + meningitis	*K. pneumoniae* (2 mg/L)	1 mg q12h; 5 mg q12h; 10 mg q12h	1 mg bid: 23 days; 5 mg bid: 19 d; 10 mg bid: 39 days	1 mg IT dose: 50 mg bid 5 mg IT dose: 45 mg bid; 10 mg IT dose: 40 mg bid	n.a.	Discharged to rehab centre	none
2019 [109]	17 y/M	Postoperative intracerebral infection	*A. baumannii* (0.5 mg/L)	4 mg q12h (drain closed 2 h) IVT; After 4 days: 4 mg q24h IT	Total: 34	47.5 mg q12h	n.a.	M, C	none
2020 [110]	56 y/M	Ventriculitis	*A. baumannii* (0.5 mg/L)	2 mg q12h (clamped 4 h); After 21 days increased to 4 mg q12h	14 in total	100 mg q12h	n.a.	3 days after increased dose: M. Discharged to ward	8 h after first IVT dose, myoclonic seizures for 4 min.
2020 [111]	28 y/M	Intracerebral infection	*A. baumannii* (0.5 mg/L)	5 mg q24h IL	9	100 mg q12h	n.a.	Spinal arachnoiditis was resolved at 12-month follow-up; Infection was cured	Spinal arachnoiditis after 9 IL doses
2020 [112]	38 y/M	Ventriculitis	*K. oxytoca* (1 mg/L)	5 mg/24 h (clamped 2 h)	11	no	D + 2 h: 178.9/310.1 mg/L D + 6 h: 35.1/41.3 mg/L D + 24 h: ND	M	none
2020 [113]	33 y/M	Intracranial infection	*A. baumannii* (0.5 mg/L)	5 mg q12h	14	100 mg q12h	n.a.	Unknown	Reduced liver function after 7 days.
2022 [114]	57 y/M	Ventriculitis	*K. pneumoniae* (2 mg/L)	3 mg q12h (clamped 2 h)	46	100 mg q12h	n.a.	M, C	none

D: Dose; mo: month; IL: intralumbar; ND: not determined; n.a.: not available; M: microbiological cure; C: clinical cure; ECOFF: epidemiological cut-off; AB: antibiotic; CSF: cerebrospinal fluid; M: male; F: female; IT: intrathecal. Doses were administered via IVT injection, unless stated otherwise.

**Table 4:** Cases with IT daptomycin.

Year (Ref.)	Age/Sex	Diagnosis	Pathogen (ECOFF)	IT Dose	Number IT Doses	Accompanying IV Dose of Same AB	Concentration in CSF	Outcome (M/C)	Side Effects
2008 [125]	62 y/M	Ventriculitis	*E. faecalis* (4 mg/L)	10 mg every third day Second episode: 5 mg every third day	First episode: 5 Second episode: 10	1 g 1 dd	23 mg/L (through, after 10 mg dose) 483 mg/L (peak; after 10 mg dose) 9.9 mg/L (through; 5 mg dose) 139 mg/L (peak, 5 mg dose)	Treatment for 2 weeks: M, C. But, relapse after 28 days. Treatment for 4 weeks: M, C and no relapse.	Transient pyrexia after each installation of daptomycin
2012 [126]	52 y/F	Ventriculitis	Coagulase negative staphylococcus (1 mg/L)	10 mg for first 2 days and then 10 mg every other day	4	10 mg/kg 1 dd	6.3 mg/L (peak) 1.39 mg/L (through)	M, C	none
2012 [127]	59 y/F	Meningitis	*E. faecium* (8 mg/L)	5 mg every 72 h IT	7	yes	n.a.	M	none
2012 [128]	64 y/M	Ventriculitis	*E. faecium* (8 mg/L)	5 mg 1 dd IT	7	no	Different concentration from right and left EVD. Right: peak 112.2 mg/L and through 1.34 mg/L. Left: peak 37.4 mg/L and 0.37 mg/L. Accumulation after 3 days	M	none
2014 [122]	23 y/M	Ventriculostomy-associated meningitis	*S. epidermidis* (1 mg/L)	5 mg once daily for 3 days and then 5 mg every 72 h	9	750 mg 1 dd	n.a.	M, C	Infusion over 4 min in 5 mL NS not tolerated. Infusion over 4 min in 3 mL NS was tolerated.
2014 [129]	19 y/F	Ventriculitis	*Enterococcus* spp. (4–8 mg/L)	5 mg every 48 h	47	8 mg/kg 1 dd	n.a.	M	none
2019 [124]	45 y/M	Subdural infection	*E. faecium* (8 mg/L)	2 doses of 5 mg at both subdural sites, and after 72 h, a dose of 2.5 mg, subdural	3	12 mg/kg	n.a.	M	none
2022 [123]	30–40 y/M	Ventriculitis	*E. faecium* (8 mg/L)	10 mg 1 dd for 3 days	3	no	n.a.	M, C	none

n.a.: not available; M: microbiological cure; C: clinical cure; IT: intrathecal; NS: normal saline; ECOFF: epidemiological cut-off; AB: antibiotic; CSF: cerebrospinal fluid; M: male; F: female. Doses were administered via IVT injection, unless stated otherwise.

**Table 5:** Summary of antibiotics with molecular weight, protein binding, hydrophylic or lipophilic nature and overview of IT doses used in clinical cases.

Antibiotic	MW (g/mol)	Approx. Serum PB (%)	Lipophilic/Hydrophylic	Number of Cases	Sources	IT Doses
Benzylpenicillin	334.4	60	Hydrophylic	many	Cases	Usually 6 mg q24h
Ampicillin	349.4	15–20	Hydrophylic	1	Case	20–40 mg q12h
Cephaloridine	415.5	10	Hydrophylic	56		5–100 mg q24h
Ceftazidime	546.6	0–20	Hydrophylic	2	Cases	10–20 mg 2×/wk or 20 mg 1×/wk
Ceftriaxone	554.6	80–95	Hydrophylic	1	Case	Intended 8 mg
Cefotiam	525.6	40	Hydrophylic	1	Case	n.a.
Meropenem	383.5	2	Hydrophylic	14 (popPK) 43 (retrosp.)	PopPK model; Retrospective study	10 mg q12h 20 mg q12h
Linezolid	337.3	30	Mod. lipophylic	1	Case	10 mg q24h
Tigecycline	585.7	70–90	Mod. lipophylic	15	Cases	Recent cases mostly used 3–5 mg q12h
Rifampicin	822.9	70–90	Lipophylic	3	Cases	5 mg q24h
Levofloxacin	361.4	20–40	Lipophylic	2	Cases	1.5–2 mg 1–3×/wk
Chloramphenicol	323.1	50	Lipophylic	3	Cases	100–750 µg q24h or 25 mg q24h
Daptomycin	1621	90–95	Hydophylic core lipophilic tail	8	Cases	5–10 mg q24–72h
Amikacin *	585.6	<10	Hydrophylic	n.a.	n.a.	30 mg q24h (R:5–100 mg q24–48h)
Tobramycin *	467.5	0	Hydrophylic	n.a.	n.a.	5–10 mg q24h (R:5–50 mg q24h)
Gentamicin *	477.6	0	Hydrophylic	n.a.	n.a.	4–10 mg q24h (R:1–20 mg q24h)
Colisitin *	~1155	60–80	Mixture **	n.a.	n.a.	10 mg q24h (R:1.6–40 mg q24h)
Polymyxin B *	~1203	60–95	Mixture **	n.a.	n.a.	5 mg q24h
Vancomycin *	1449	0–100	Hydrophylic	n.a.	n.a.	10–20 mg q24h (R:5–50 mg q24h)
Teicoplanin *	1880	95	Mod. Lipophylic	n.a.	n.a.	5–20 mg q24h

MW: molecular weight; PB protein binding; IT: intrathecal; mod: moderately; R: range. *: these antibiotics were not included in this review, but they were added for completeness based on a previous review [8]. ** mixture of hydrophylic/lipophylic groups.

## Data Availability

Not applicable.
